# Exogenous Putrescine Modulates Nitrate Reductase-Dependent NO Production in Cucumber Seedlings Subjected to Salt Stress

**DOI:** 10.3390/metabo13091030

**Published:** 2023-09-21

**Authors:** Natalia Napieraj, Małgorzata Janicka, Beata Augustyniak, Małgorzata Reda

**Affiliations:** 1 Department of Plant Molecular Physiology, Faculty of Biological Science, University of Wrocław, Kanonia 6/8, 50-328 Wrocław, Poland; natalia.napieraj2@uwr.edu.pl (N.N.); malgorzata.janicka@uwr.edu.pl (M.J.); 2 Department of Genetic Biochemistry, Faculty of Biotechnology, University of Wrocław, Przybyszewskiego 63/77, 51-148 Wrocław, Poland; beata.augustyniak@uwr.edu.pl

**Keywords:** putrescine, polyamines, nitric oxide, nitrate reductase (NR), nitric oxide synthase-like activity, salt stress

## Abstract

Polyamines (PAs) are small aliphatic compounds that participate in the plant response to abiotic stresses. They also participate in nitric oxide (NO) production in plants; however, their role in this process remains unknown. Therefore, the study aimed to investigate the role of putrescine (Put) in NO production in the roots of cucumber seedlings subjected to salt stress (120 mM NaCl) for 1 and 24 h. In salinity, exogenous Put can regulate NO levels by managing NO biosynthesis pathways in a time-dependent manner. In cucumber roots exposed to 1 h of salinity, exogenous Put reduced NO level by decreasing nitrate reductase (NR)-dependent NO production and reduced nitric oxide synthase-like (NOS-like) activity. In contrast, during a 24 h salinity exposure, Put treatment boosted NO levels, counteracting the inhibitory effect of salinity on the NR and plasma membrane nitrate reductase (PM-NR) activity in cucumber roots. The role of endogenous Put in salt-induced NO generation was confirmed using Put biosynthesis inhibitors. Furthermore, the application of Put can modulate the NR activity at the genetic and post-translational levels. After 1 h of salt stress, exogenous Put upregulated *CsNR1* and *CsNR2* expression and downregulated *CsNR3* expression. Put also decreased the NR activation state, indicating a reduction in the level of active dephosphorylated NR (dpNR) in the total enzyme pool. Conversely, in the roots of plants subjected to 24 h of salinity, exogenous Put enhanced the NR activation state, indicating an enhancement of the dpNR form in the total NR pool. These changes were accompanied by a modification of endogenous PA content. Application of exogenous Put led to an increase in the amount of Put in the roots and reduced endogenous spermine (Spm) content in cucumber roots under 24 h salinity. The regulatory role of exogenous Put on NO biosynthesis pathways may link with plant mechanisms of response to salt stress.

## 1. Introduction

It is estimated that approximately 90% of all arable lands are exposed to environmental stresses, such as drought, low or high temperatures, salt stress, and others [1]. Among them, salt stress seems to be the most threatening. It is predicted that more than 50% of arable land will be salinized by 2050 [2]. Salinity can reduce the ability of roots to uptake water and lead to the inhibition of several physiological and biochemical processes. Prolonged exposure to NaCl can result in ionic toxicity, osmotic stress, and, in consequence, nutritional and oxidative disorders. The accumulation of reactive oxygen species (ROS) leads to lipid peroxidation, protein oxidation, and inhibition of enzyme activities [3]. As a result, processes such as germination, growth, and development are affected, and plant productivity is notably reduced [3,4].

Fortunately, plants developed strategies to cope with salt stress. They can improve tolerance to salinity by modulating numerous biochemical pathways, inducing hormones and signaling molecules, and leading to the activation of stress-responsive genes [5]. A multi-purpose gaseous molecule, nitric oxide (NO), is involved in plant responses to environmental stress [6]. As a signaling molecule, NO can modify proteins at the post-translational level, interact with phytohormones, regulate gene expression, and participate in the production of certain second messengers, activating plant responses to salinity [3,7,8]. Previous studies have demonstrated that the treatment of NO donors alleviated the adverse effects of salt stress in some plant species [9,10,11]. Therefore, NO application in agriculture is considered, for example, by using special carriers for NO delivery [12]. The modulation of NO biosynthesis in plants at the molecular and genetic levels is also considered. For this reason, complete knowledge of NO biosynthesis is necessary.

The pathways of NO generation in plants are still not fully understood [13]. The possible substrates for the oxidative mechanism of NO production are L-arginine via nitric oxide synthase-like (NOS-like) activity, polyamines (PAs), and hydroxylamines (HAs) [14,15,16]. In reductive pathways, NO is produced as a result of nitrite reduction by cytosolic nitrate reductase (NR), plasma membrane-bound nitrite-NO reductase (NiNOR), mitochondrial nitrite reduction, and other molybdoenzymes, like sulfite oxidase (SO), amidoxime-reducing component (ARC), aldehyde oxidase (AO), and xanthine oxidoreductase (XOR) [17,18,19,20]. Among these potential NO production pathways, two of them, involving NR and NOS-like activity, seem to be the most described and explored. The main role of NR in plants is a reduction of nitrate to nitrite during nitrate assimilation. However, NR can reduce nitrite to NO using nicotinamide adenine dinucleotide reduced form (NADH) as an electron donor in specific conditions [21,22]. Some earlier and recent studies have indicated the participation of NR in NO production in plants during stomata closure, plant–bacteria symbiosis, and gravitropic response [23,24,25]. Furthermore, NR-dependent NO formation was reported during plant response to abiotic and biotic stresses, like pathogen attack, water stress, low-temperature stress, heavy metal stress, and salt stress [26,27,28,29]. Besides cytosol, NR is also localized in the plasma membrane. The role of plasma membrane nitrate reductase (PM-NR) is the reduction of NO_3_^−^ to NO_2_^−^; then, NiNOR catalyzes the reduction of apoplastic NO_2_^−^ to NO in plant roots during anoxia [29,30]. The studies showed that NiNOR may take part in NO generation in mycorrhizal tobacco roots [31], whereas PM-NR may participate in NO production in cucumber roots under salt stress [29]. Another potential NO pathway in plants includes NOS-like activity. In animals, nitric oxide synthase (NOS) catalyzes the nicotinamide adenine dinucleotide phosphate, reduced form (NADPH)-dependent five-electron oxidation of L-arginine to L-citrulline and NO [6]. In plants, the direct molecular and genetic evidence for the presence of NOS is still missing [32]. However, some studies demonstrated the activity of NOS-like enzymes in plant roots, leaves, and cell cultures, as well as described a reduction of NO levels in plants treated with inhibitors of animal NOS [33,34].

As mentioned above, PAs are considered a possible route of NO production in plants. PAs are low-molecular-weight aliphatic amine compounds that participate in multiple physiological processes in plants. The most common PAs in plants are putrescine (Put), spermidine (Spd), and spermine (Spm) [35]. The protective role of PAs in plant tolerance to abiotic stresses, such as salt stress, drought, high and low temperatures, and heavy metal stress, has been confirmed in numerous plant species [4,36,37]. PAs can modify plant tolerance to stress by interacting with plant hormones, like abscisic acid (ABA), ethylene, or gibberellins [35,38,39]. They have an additive impact on DNA, the membrane, and molecule stability, and participate in plant processes, such as RNA processing, maintenance of DNA structure, translation, and others [40], and also regulate the expression of several genes related to plant response to stress, photosynthesis, and ion transport [41,42]. Furthermore, PAs are closely combined with two essential signaling molecules: hydrogen peroxide (H_2_O_2_), a product of PA catabolism, and NO [15,43]. The role of potential crosstalk between PAs and NO in plant adaptation to stress has been discussed in recent years. Some evidence suggests a role of PAs in NO biosynthesis, which may be a link between the PA-mediated stress response and other stress mediators [3]. It has been proposed that PAs can induce NO production in some plant species, such as *Arabidopsis*, wheat, and cucumber [15,44,45]. However, this issue is still largely unexplained. Wimalasekera et al. [46] suggested that PA catabolism may be related to NO generation. On the other hand, it has been demonstrated that the exogenous application of PAs modulated the NO production in plants under abiotic stresses through the modification of NR and/or NOS-like pathways [35,36,45].

Cucumber (*Cucumis sativus* L.) is an economically important vegetable whose genome has been sequenced [47,48]. Due to the small number of genes, rich diversity of sex expression, and short life cycle, the cucumber is developed as a new model plant [49]. However, cucumber is moderately sensitive to salt stress, especially at germination and seedling stages [50]. Therefore, the main goal of our study was to investigate the influence of Put on two pathways of NO production via NR and NOS-like activity in cucumber seedlings subjected to moderate salt stress (120 mM NaCl). According to our knowledge, only a few studies have revealed PA-dependent NO production in plants under salt stress.

## 2. Materials and Methods

### 2.1. Plant Material and Treatment

Cucumber (*Cucumis sativus* L. var. Wisconsin) seedlings were used in all experiments. This is one of the oldest varieties developed at the University of Wisconsin. There were no problems with cultivation. It was characterized by multiple-disease resistance [51]. For this reason, the variety has been used in many of our previous studies [29,50,52,53]. Cucumber seeds were germinated in the dark on wet filter paper for 48 h at 27 °C. Then, seedlings were directly transferred to a nutrient solution, pH 6.5, containing 1.7 mM KNO_3_, 1.7 mM Ca(NO_3_)_2_, 0.33 mM KH_2_PO_4_, 0.33 mM MgSO_4_, and microelements: 25 μM ferric citrate, 3.33 μM MnSO_4_, 1.7 μM H_3_BO_3_, 0.3 μM CuSO_4_, 0.003 μM ZnSO_4_, and 0.017 μM Na_2_MoO_4_ (control). All reagents used for the nutrient solution were from Chempur (Chempur, Piekary Śląskie, Poland). Plants were grown hydroponically in a 16 h photoperiod (180 μmol m^−2^ s^−1^) at 23 °C during the day and 22 °C at night. Before harvest, the nutrient solution was replaced by a new one for 1 or 24 h. The new nutrient solution was supplemented with additional compounds with a final concentration of 120 mM NaCl, 1 mM Put, 1 mM Spd, 1 mM Spm, 0.1 mM difluoromethylornithine (DFMO), 0.1 mM difluoromethylarginine (DFMA), 0.1 mM sodium tungstate (WO) or 0.1 mM N^G^-nitro-L-arginine methyl ester (L-NAME). DFMO was used as an inhibitor of ornithine decarboxylase (ODC) activity, and DFMA was used as an inhibitor of arginine decarboxylase (ADC) activity [54]. Sodium tungstate is an inhibitor of molybdoenzymes (NR, SO, ARC, AO, XOR), and L-NAME was used as an inhibitor of NOS-like activity [35,55]. Five or six days after germination, the salt stress was started (for 24 h and 1 h NaCl treatment, respectively). All the above reagents were from Sigma (Sigma-Aldrich, St. Louis, MO, USA). Inhibitors of ODC and ADC were from Cayman Chemical (Cayman Chemical, Ann Arbor, MI, USA). Six-day-old seedlings were collected for analysis. Roots were harvested after 6 h of starting illumination. Basic plant growth parameters, such as shoot and root length, fresh shoot and root weight, and relative water content (RWC) [56,57], were determined. 

### 2.2. Fluorescent Detection of Endogenous NO

The amount of NO in root tissues was detected using fluorescent NO indicator dye, 5,6-diaminofluorescein diacetate (DAF-2 DA) [58]. Roots were incubated for 10 min in 20 mM Hepes-KOH (pH 7.4), containing 10 µM DAF-2 DA (Sigma-Aldrich, St. Louis, MO, USA) in the dark at room temperature, then washed twice for 10 min in fresh Hepes-KOH buffer to remove the excess of the fluorophore and mounted on a microscope slide in the same buffer. The NO-associated fluorescence was detected using a Zeiss Axio Image M2 fluorescent microscope (Carl Zeiss Microscopy GmbH, Jena, Germany) and a Tag-YFP filter with emission at 524 nm. The intensity of NO-dependent green fluorescence was examined using Adobe Photoshop CC software (24.7, Adobe, San Jose, CA, USA) and expressed as the average intensity value in the green channel [29]. 

### 2.3. Colorimetric Measurement of NO Level

The content of NO was also measured colorimetrically with Griess reagent as described by Filippou et al. [59] with some modifications. Fresh root samples were ground in a chilled mortar with ice-cold 50 mM acetate buffer (pH 3.6, containing 4% (*w*/*v*) zinc acetate (Chempur, Piekary Śląskie, Poland). The homogenate was centrifuged at 10,000× *g* and 4 °C for 15 min. The supernatant was mixed with Griess reagent, incubated at room temperature for 30 min, and absorbance was measured at 540 nm. The level of NO was calculated by comparison with the standard NaNO_2_ curve. The Griess reagent contained 1% sulphanilamide (Sigma-Aldrich, St. Louis, MO, USA) in hydrochloric acid and 0.01% N-(1-Naphthyl)ethylenediamine dihydrochloride (NED, Sigma-Aldrich, St. Louis, MO, USA) mixing in the proportion 1:1.

### 2.4. Cytosolic Nitrate Reductase Activity

Nitrate reductase (EC 1.7.1.1) activity was determined according to Kaiser and Huber [60] with some modifications. Frozen root samples were ground in a chilled mortar with the extraction buffer containing 50 mM Hepes-KOH (pH 7.5), 1 mM DTT, 1% BSA, 0.5 mM PMSF, and 1% (*w*/*v*) PVPP, and then centrifuged. Buffer reagents were from Sigma (Sigma-Aldrich, St. Louis, MO, USA) and BioShop (BioShop Canada Inc., Burlington, ON, Canada). NR occurs in three states: unphosphorylated NR; phosphorylated NR (pNR), which are active forms; and pNR in a complex with 14-3-3 protein (pNR:14-3-3), which is inactive [21]. NR activity was measured in the absence or presence of Mg^2+^ (total NR activity and actual NR activity, respectively). The actual NR activity measured in the presence of Mg^2+^ shows the activity of dephosphorylated form of enzyme (dpNR), whereas total NR activity determined in the presence of EDTA (Chempur, Piekary Śląskie, Poland) represents the activity of both unphosphorylated and phosphorylated forms of NR. In addition, the NR activation state was determined. This parameter was expressed as a percentage value of the actual/total activity ratio and informed about the level of dephosphorylated NR in relation to the total NR content [21]. The reaction mixture contained 50 mM Hepes-KOH (pH 7.5), 10 mM KNO_3_, 5 mM EDTA (total NR activity), or 5 mM MgCl_2_ (actual NR activity). The reaction was started by the addition of 0.2 mM NADH (Sigma-Aldrich, St. Louis, MO, USA) and stopped by the addition of 1 M zinc acetate. After centrifugation, the amount of nitrite was measured calorimetrically at 540 nm [61]. Figure S1 in Supplementary Materials represents the graphical illustration of total and actual NR activities, as well as NR activation state. 

### 2.5. Plasma Membrane Isolation and Determination of Plasma Membrane Nitrate Reductase Activity

The plasma membrane vesicles were isolated from cucumber roots using a two-phase system according to the method of Larson [62] with some modifications described by Kłobus [63]. The purity of the PM fraction was verified by measuring the activities of two cytosol marker enzymes—phosphoenolpyruvate carboxylase (PEPC) and alcohol dehydrogenase (ADH)—according to the method of Spalding and Edwards [64] and Chung and Ferl [65], and presented in Supplementary Materials (Table S1). The protein content of the PM fraction was determined according to Bradford [66]. The PM fraction was used to measure the PM-NR activity. The reaction mixture contained 50 mM Hepes-KOH (pH 7.5), 10 mM KNO_3_, 1% Triton X-100, and 5 mM EDTA, and it was started by the addition of 0.2 mM NADH and stopped by 1 M zinc acetate. After centrifugation, the amount of nitrite was measured calorimetrically at 540 nm [61]. The reagents used in the analysis were from Chempur (Chempur, Piekary Śląskie, Poland) and Sigma (Sigma-Aldrich, St. Louis, MO, USA).

### 2.6. Expression of CsNR Genes

Real-time PCR with the LightCycler 2.0 system (Roche, Basel, Switzerland) was used to assess the expression of genes encoding nitrate reductase in cucumber: *CsNR1* (GenBank (National Library of Medicine, Bethesda, MD, USA) accession number HM755943.1), *CsNR2* (HM755944.1), and *CsNR3* (HO116134.1). The gene encoding TIP41-like protein (GW881871.1) was used as a reference to standardize the results due to its expression stability in cucumber roots [67]. Total RNA was extracted from cucumber seedling roots with EXTRAzol (Blirt, Gdańsk, Poland) following the manufacturer’s instructions. The concentration and purity of RNA were measured spectrophotometrically (NanoDrop ND-1000, Thermo Fisher Scientific, Waltham, MA, USA). The samples with the 260/280 ratio between 1.9 and 2.1 were used for cDNA synthesis. To avoid any DNA contamination, RNA samples were treated with RNase-free DNase I (Fermentas, Waltham, MA, USA) for 30 min at 37 °C. The reaction was stopped by adding 2.5 mM EDTA and incubating at 65 °C for 10 min. The samples were then reverse-transcribed into the first-strand cDNA using the High-Capacity cDNA Reverse Transcription Kit (Applied Biosystems, Waltham, MA, USA) according to the manufacturer’s protocol. In the *CsNR* expression experiment, cDNA was used with the Real-Time 2× PCR Master Mix SYBR^®^ kit (A&A Biotechnology, Gdańsk, Poland). The following conditions were applied: 30 s at 95 °C, 40 cycles of 10 s at 95 °C, 10 s at 56 °C, and 12 s at 72 °C, with final melting for 15 s at 65 °C. The specific *CsNR1–3* primers, according to Reda et al. [52], and TIP41-like protein primers, according to Migocka and Papierniak [67], were used. 

### 2.7. NOS-like Activity

The NOS-like activity was measured according to Sun et al. [28] with some modifications. Cucumber roots were homogenized in a chilled mortar with addition of 100 mM Hepes-KOH (pH 7.5) containing 1 mM EDTA, 5 mM DTT, 0.5 mM PMSF, 10% glycerol, 0.1% Triton X-100, 20 μM FAD, and 1% PVPP. A homogenate was centrifuged at 13,000× *g* for 20 min at 4 °C. Obtained supernatant was used for further analysis. The reaction mixture contained 100 mM K_2_HPO_4_–KH_2_PO_4_ (pH 7.0, 1 mM L-arginine, 2 mM MgCl_2_, 0.3 mM CaCl_2_, 4 μM BH_4_, 1 μM FAD, 1 μM FMN, 0.2 mM DTT, and 0.2 mM NADPH. NOS-like activity was expressed as NADPH consumption during the reaction and monitored spectrophotometrically by measuring the decrease in absorbance at 340 nm for 3 min. Results were calculated using the extinction coefficient of NADPH (ε = 6.22 mM^−1^ cm^−1^). The reagents used for extraction and reaction mixture were from Chempur (Chempur, Piekary Śląskie, Poland) and from Sigma (Sigma-Aldrich, St. Louis, MO, USA).

### 2.8. Endogenous PA Content

The isolation of free PAs was performed according to Imai et al. [68] with some modifications. Cucumber roots were extracted with 4% (*w*/*v*) perchloric acid (PCA) for 1 h at 4 °C. The homogenate was centrifuged at 48,000× *g* for 20 min at 4 °C, and after centrifugation, the supernatant containing free PAs was collected. For PA dansylation, 0.2 mL of saturated sodium carbonate (Merck, Darmstadt, Germany) and 0.4 mL of dansyl chloride (5 mg/mL in acetone, Chempur, Piekary Śląskie, Poland) were added to 0.2 mL of sample. After brief vortexing, the mixture was incubated in darkness for 1 h at 60 °C. To remove excess dansyl reagent (Merck, Darmstadt, Germany), 0.2 mL of proline (150 mg/mL, Pol-Aura, Zawroty, Poland) was added. Dansylated PAs were extracted by 0.5 mL of toluene (Chempur, Piekary Śląskie, Poland), dried under nitrogen flow, re-suspended in 0.2 mL of acetonitrile (VWR Chemicals, Leuven, Belgium). The samples were analyzed using a Waters Acquity UPLC system with a 2996 PDA detector on an Acquity UPLC HSS T3 2.1 × 100 mm 1.8 μm column. The mobile phase was A = water and B = acetonitrile, in a gradient flow: initial: 40% A/60% B; 1.17 min: 40% A/60% B; 1.65 min: 30% A/70% B; 4.0 min: 25% A/75% B; 7.57 min: 0% A/100% B; 8.00 min: 0% A/100% B; 8.5: 40% A/60% B; 11.5 min: 40% A/60% B with a 0.4 mL/min flow rate. The peak integration was at 366 nm. The results were standardized with mixtures of dansylated polyamine standards (Put, Spd, Spm). 

### 2.9. Statistics

All data shown in the figures are means ± SE (standard error) of at least 3 independent experiments. The LightCycler software 4.1 (Roche, Basel, Switzerland) was used to analyze real-time PCR data. The results were statistically analyzed using the Student’s *t*-test. Significance was evaluated at *p* < 0.1 (*), *p* < 0.05 (**), and *p* < 0.001 (***). Asterisks indicate a significant difference between treatments. 

## 3. Results

### 3.1. Effect of Put and/or NaCl on NO Production in Cucumber Roots

**Figure 1:**
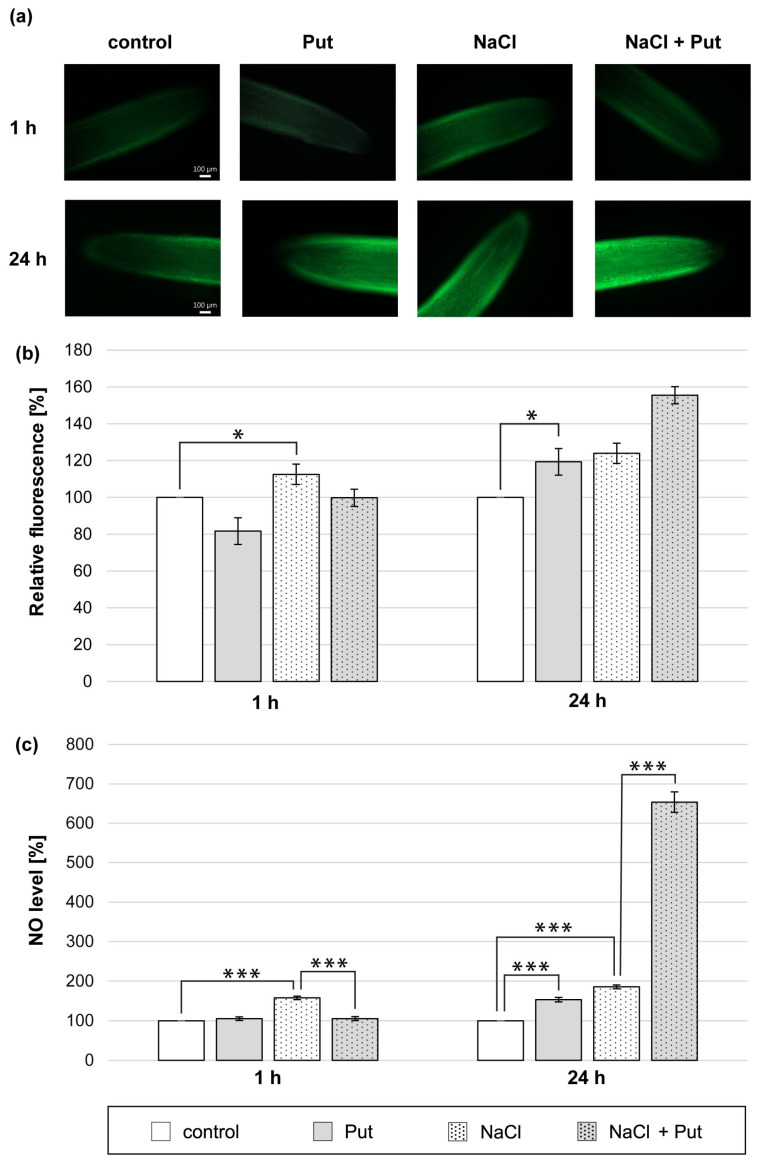
The level of NO in roots of cucumber treated with 1 mM Put and/or 120 mM NaCl for 1 h and 24 h. (**a**) Bio-imaging of NO generation in root apical segments of cucumbers using DAF-2 DA dye. Bar, 100 µm. (**b**) Relative efficiency of Put and/or NaCl for NO release from root apical segments of cucumber seedlings. (**c**) NO level in cucumber roots measured colorimetrically using the Griess reagent. The average value of untreated plants was 9.40 ± 2.00 nmol NO_2_^−^ × gFW^−1^. Significance was evaluated at: *p* < 0.1 (*); *p* < 0.001 (***).

We demonstrated that the Put application for 1 h did not change the NO level measured with microscopic and colorimetric methods in cucumber roots (Figure 1a–c). On the other hand, NO biosynthesis was significantly stimulated in the roots of plants subjected to NaCl for 1 h. The NO-dependent fluorescence increased by about 15% compared to unstressed plants (Figure 1a,b). The NO level measured colorimetrically in root tissue was approximately 60% higher compared to the roots of control, unstressed plants (Figure 1c). However, the co-application of Put and NaCl for 1 h prevented NaCl-related NO increase observed in roots. The NO level measured using Griess reagent was reduced by about 50% compared to salinized plants (Figure 1c). The longer, 24 h treatment of plants with Put or NaCl and Put with NaCl led to an increase in NO production in seedlings roots. Put significantly increased the NO level in roots by approximately 20% and 50%, as measured by the fluorescent and colorimetric methods, respectively (Figure 1a–c). The treatment of cucumber seedlings with NaCl for 24 h increased the NO level in roots compared to the control plants by approximately 25% and 85%, determined using the fluorescent and colorimetric methods, respectively (Figure 1a–c). Moreover, the NO production was further stimulated in the roots of plants treated with Put and NaCl for 24 h, compared to salinized plants (Figure 1a–c).

### 3.2. Effect of DFMA and DFMO on NO Production in Cucumber Roots Treated with NaCl

**Figure 2:**
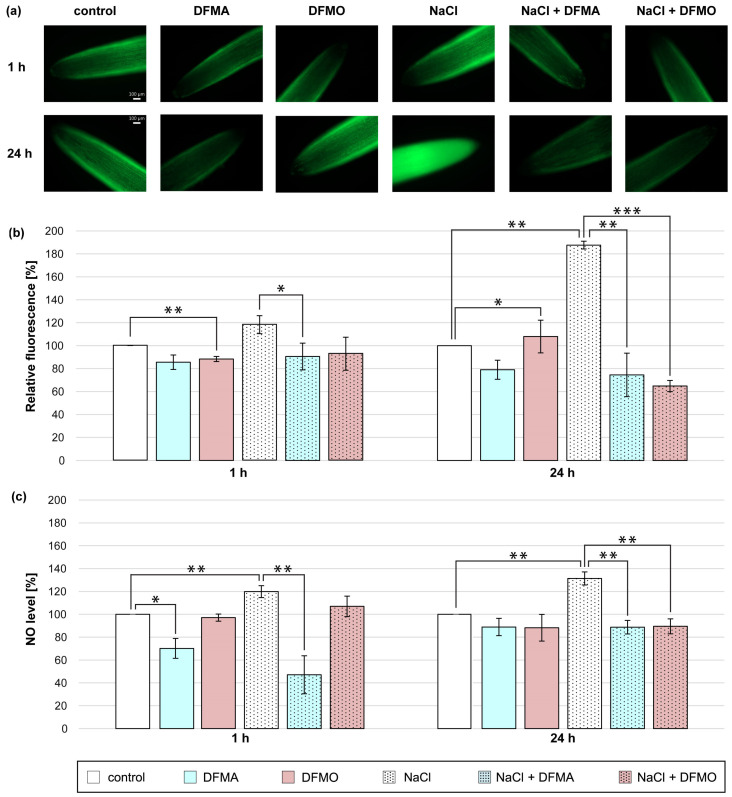
The level of NO in roots of cucumber treated with 0.1 mM DFMA, 0.1 mM DFMO, and/or 120 mM NaCl for 1 h and 24 h. (**a**) Bio-imaging of NO generation in root apical segments of cucumbers using fluorescent method with DAF-2 DA dye. Bar, 100 µm. (**b**) Relative efficiency of DFMA, DFMO, and/or NaCl for NO release from root apical segments of cucumber seedlings. (**c**) NO level in cucumber roots measured using the Griess reagent. The average value of untreated plants was 5.43 ± 0.70 nmol NO_2_^−^ × gFW^−1^. Significance was evaluated at: *p* < 0.1 (*); *p* < 0.05 (**); *p* < 0.001 (***).

In the present study, cucumber seedlings were treated with NaCl and DFMA or DFMO, inhibitors of ADC and ODC pathway, respectively, to verify the involvement of endogenous Put in NO production in roots of plants exposed to salt stress (Figure 2). A short-term (1 h) salt stress treatment stimulated the NO production in the cucumber roots, but both inhibitors used reduced NO generation in NaCl-treated plants. The NO amount, expressed as NO-dependent green fluorescence, and the NO level measured colorimetrically, were decreased in roots of plants treated with NaCl and DFMA by approximately 30% and 70%, respectively, compared to salt-stressed plants (Figure 2a–c). Similarly, the application of DFMA or DFMO significantly reduced the NO biosynthesis in the roots of plants treated with NaCl for 24 h. The observed decreases in NO levels after both inhibitor treatments were more pronounced using fluorescence than the colorimetric method. NO-dependent DAF-2 DA fluorescence dropped by 110–120% in the roots of plants treated with DFMA and DFMO in the presence of NaCl (Figure 2a–c).

### 3.3. Effect of Sodium Tungstate and L-NAME on NO Production in Cucumber Roots Treated with NaCl

**Figure 3:**
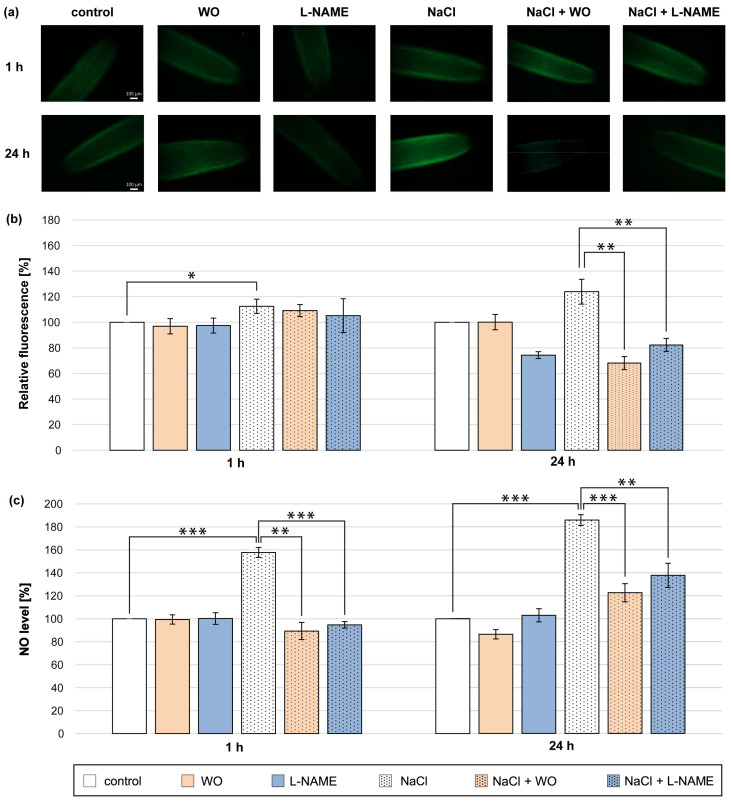
NO level in roots of cucumber treated with 0.1 mM WO, 0.1 mM L-NAME, and/or 120 mM NaCl for 1 h and 24 h. (**a**) Bio-imaging of NO generation in root apical segments of cucumbers using a fluorescent method with DAF-2 DA dye. Bar, 100 µm. (**b**) Relative efficiency of WO, L-NAME, and/or NaCl for NO release from root apical segments of cucumber seedlings. (**c**) NO level in cucumber roots measured using the Griess reagent. The average value of untreated plants was 9.08 ± 1.63 nmol NO_2_^−^ × gFW^−1^. Significance was evaluated at: *p* < 0.1 (*); *p* < 0.05 (**); *p* < 0.001 (***).

The best-known protein in plant cells that participates in NO biosynthesis is NR [17]. Therefore, we decided to check whether the increase in NO level during NaCl application could be due to the participation of NR in this process. For this purpose, WO, a commonly used NR inhibitor, was applied. Plants were treated with NaCl and sodium tungstate for 1 and 24 h (Figure 3). 

The presence of tungstate effectively prevented the increase in NO level in the roots of plants treated with NaCl for 1 h. The inhibitory effect of tungstate was significantly pronounced in the colorimetric method (Figure 3c). Tungstate also significantly prevented a higher production of NO in the roots of plants during the 24 h NaCl treatment. The reduction of NO level in the roots of plants incubated for 24 h in the presence of tungstate and NaCl was about 55–60% compared to plants incubated only with salt and was expressed by using both methods (Figure 3a–c). Further, the inhibitor of animal NOS (L-NAME) was introduced to the medium to investigate the potential role of NOS-like activity in NO production in cucumber roots under salt-stress conditions. The co-application of NaCl and L-NAME for 1 h caused a reduction in NO level measured colorimetrically by approximately 70% compared to plants treated only with NaCl (Figure 3c). Furthermore, L-NAME led to a reduction in NO amount detected in roots treated with salt for 24 h. The decrease in NO level in salt-treated roots in the presence of L-NAME was about 40–50% and was similarly pronounced by fluorescent and colorimetric methods (Figure 3a–c).

### 3.4. Effect of Put and/or NaCl on Nitrate Reductase Activity in Cucumber Roots

**Figure 4:**
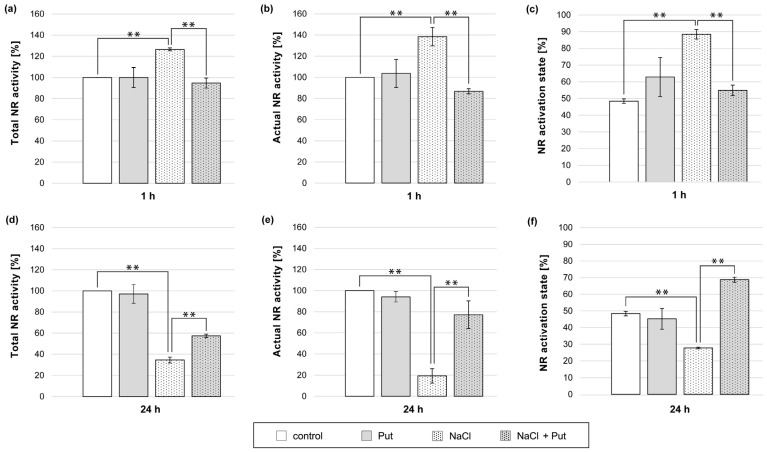
Total activity, actual activity, and activation state of NR in roots of cucumber seedlings treated with 1 mM Put and/or 120 mM NaCl for (**a**–**c**) 1 h and (**d**–**f**) 24 h. The average value of control plants, based on the results of individual experiments, was 0.85 ± 0.16 µmol NO_2_^−^ × gFW^−1^ × h^−1^ for total NR activity (**a**,**d**) and 0.36 ± 0.13 µmol NO_2_^−^ × gFW^−1^ × h^−1^ for actual NR activity (**b**,**e**). Significance was evaluated at: *p* < 0.05 (**).

Since sodium tungstate (NR inhibitor) abolished the stimulating effect of NaCl on NO production in cucumber seedlings (Figure 3), the activity of NR in plants treated with Put and NaCl was determined (Figure 4). As mentioned earlier, NR occurs in three states. Free, unphosphorylated NR is active and responsible for NO_3_^−^ reduction. When NR occurs in its phosphorylated form (pNR), the enzyme is still active until the 14-3-3 protein recognizes and binds to pNR in the presence of divalent cations (e.g., Mg^2+^) or PAs [21]. During the estimation of actual NR activity in the presence of Mg^2+^ in the reaction mixture, the (pNR) interacts with 14-3-3 and forms inactive pNR:14-3-3 complexes. Hence, actual activity describes the activity of unphosphorylated NR (dpNR) only (Figure S1a). To determine the total activity of NR, EDTA (Mg^2+^ chelator) was added to the reaction mixture. In these conditions, inactive pNR:14-3-3 complexes were not present, and the activity of both unphosphorylated and phosphorylated forms of NR was measured (Figure S1b) [21]. 

Treatment of plants with 1 mM Put for 1 h did not cause significant changes in total and actual NR activities or NR activation state in cucumber roots (Figure 4a–c). In contrast, the exposure of plants to NaCl for 1 h significantly increased both total and actual NR activities in roots by approximately 25% and 40%, respectively (Figure 4a,b). The NR activation state in the same roots was 40% higher than that in the roots of untreated control plants (Figure 4c). In plants treated simultaneously with Put and NaCl for 1 h, the total and actual NR activities in roots decreased by approximately 30% and 50%, respectively, compared to both NR activities detected in the roots of NaCl-treated plants (Figure 4a,b). Furthermore, in salt-stressed plants, Put treatment reduced the NR activation state compared to the NR activation state in the roots of salt-stressed plants (Figure 4c). Longer (24 h) treatment of seedlings with Put did not change the measured NR activities and NR activation state in roots compared to untreated, control plant roots (Figure 4d–f). However, 24 h salt incubation significantly decreased the total and actual NR activity in the roots by approximately 65% and 80%, respectively (Figure 4d,e). In addition, the NR activation state was 20% lower than that of the control plant roots (Figure 4f). In turn, the simultaneous treatment of seedlings with Put and NaCl increased the total and actual NR activity measured in the roots. NR activities were 20% and 60%, respectively, higher than those measured in the roots of plants treated with NaCl (Figure 4d,e). Moreover, the NR activation state was also stimulated in the roots of Put- and NaCl-treated plants (Figure 4f). 

### 3.5. Effect of Put and/or NaCl on Plasma-Membrane Nitrate Reductase Activity in Cucumber Roots

**Figure 5:**
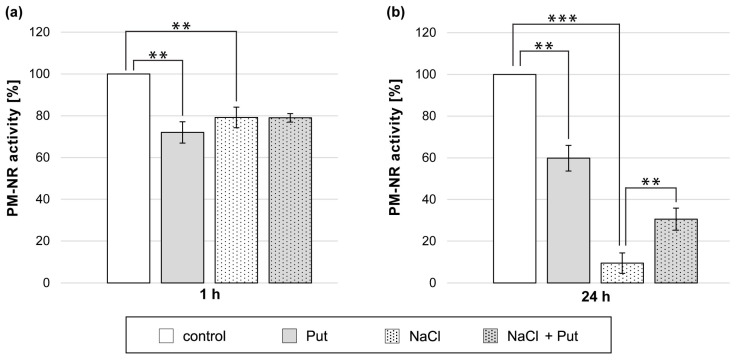
PM-NR activity in roots of cucumber seedlings treated with 1 mM Put and/or 120 mM NaCl for (**a**) 1 h and (**b**) 24 h. The average value of control plants, based on the results of individual experiments, was (**a**) 0.65 ± 0.04 µmol NO_2_^−^ × mg^−1^ × h^−1^ and (**b**) 0.18 ± 0.04 µmol NO_2_^−^ × mg^−1^ × h^−1^ for PM-NR activity. Significance was evaluated at: *p* < 0.05 (**); *p* < 0.001 (***).

Treatment of cucumber seedlings with Put or NaCl for 1 h reduced PM-NR activity in roots. The PM-NR activity was 20–30% lower than in control, untreated plant roots (Figure 5a). However, exogenous Put did not affect PM-NR activity in the roots of cucumber seedlings exposed to salt for 1 h (Figure 5a). Longer, 24 h exposition of seedlings to Put or NaCl also markedly reduced PM-NR activity in roots. The PM-NR activity was 40% lower in Put-treated plants and almost 90% lower in NaCl-treated plants compared to non-salt-stressed plants (Figure 5b). Interestingly, the simultaneous application of Put and NaCl for 24 h reduced the strong inhibitory effect of salt on that enzyme activity. In those conditions, the PM-NR activity measured in roots was 20% higher compared to plants treated only with NaCl (Figure 5b).

### 3.6. Effect of Put and/or NaCl on CsNR Expression in Cucumber Roots

**Figure 6:**
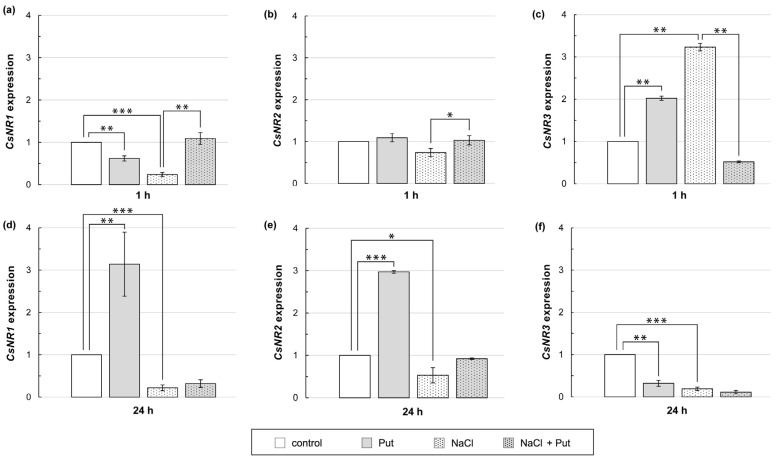
Relative expression of *CsNR* genes in roots of cucumber seedlings treated with 1 mM Put and/or 120 mM NaCl for (**a**–**c**) 1 h and (**d**–**f**) 24 h. The expression of *CsNR* genes was normalized using the TIP41-like gene as reference. Significance was evaluated at: *p* < 0.1 (*); *p* < 0.05 (**); *p* < 0.001 (***).

Exogenously applied Put and NaCl contributed to the changes in total NR activity (Figure 4). Therefore, it was examined whether Put and/or NaCl affected the expression of *CsNR* genes encoding the NR protein. Treatment of plants with Put or NaCl for 1 h reduced the *CsNR1* transcript level in roots, whereas expression of this gene increased when plants were incubated in the presence of Put together with NaCl (Figure 6a). Expression of *CsNR2* did not significantly change when plants were incubated with Put or NaCl separately, but when Put and NaCl were applied together, the expression of *CsNR2* in roots increased slightly compared to that in salt-stressed roots (Figure 6b). The expression of *CsNR3* in roots increased 2–3 fold in response to plant exposition to Put or NaCl (Figure 6c). On the contrary, when Put was applied together with salt, the expression of *CsNR3* was strongly inhibited compared to that detected in the roots of salt-stressed plants (Figure 6c). The longer, 24 h treatment of plants with Put increased *CsNR1* and *CsNR2* expression in roots approximately 3-fold and reduced *CsNR3* expression in the same time by about 70%, compared to untreated plants. In cucumbers exposed to salt stress for 24 h, the expression of all *CsNR* genes was decreased by approximately 80%, 50%, and 80%, respectively, in relation to control plants. However, Put treatment had no significant effect on the *CsNR1–3* expression in NaCl-stressed plants (Figure 6d–f).

### 3.7. Effect of Put and/or NaCl on NOS-Like Activity in Cucumber Roots

**Figure 7:**
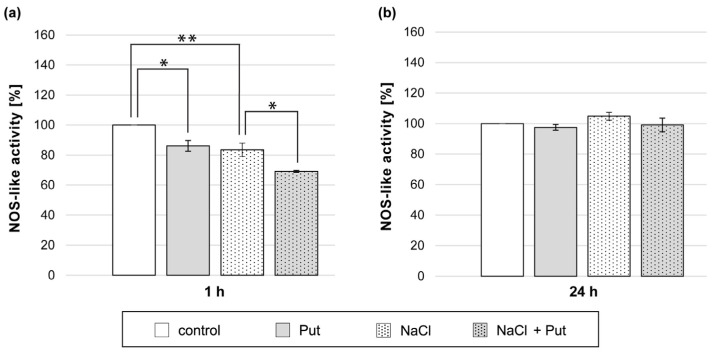
NOS-like activity in roots of cucumber seedlings treated with 1 mM Put and/or 120 mM NaCl for (**a**) 1 h and (**b**) 24 h. The average value of control plants, based on the results of individual experiments, was 11.75 ± 2.10 nmol NADPH × mg^−1^ × min^−1^. Significance was evaluated at: *p* < 0.1 (*); *p* < 0.05 (**).

Analysis of NO production in cucumber roots exposed to L-NAME and NaCl showed that the inhibitor of NOS activity successfully decreased NO levels in cucumber roots subjected to salt stress (Figure 3). Therefore, we decided to investigate the activity of NOS-like activity in cucumber roots exposed to Put and/or NaCl. The study revealed that NOS-like activity was reduced in plants treated for 1 h with Put or NaCl separately, compared to the activity measured in the roots of control seedlings (Figure 7a). Simultaneous application of Put and NaCl for 1 h resulted in a further decrease in NOS-like activity, which was 15% lower when compared to plants treated with NaCl only (Figure 7a). However, in plants treated with Put and/or NaCl for 24 h, no significant changes in NOS-like activity were observed (Figure 7b).

### 3.8. Effect of Put and/or NaCl on Endogenous PAs Level in Cucumber Roots

**Figure 8:**
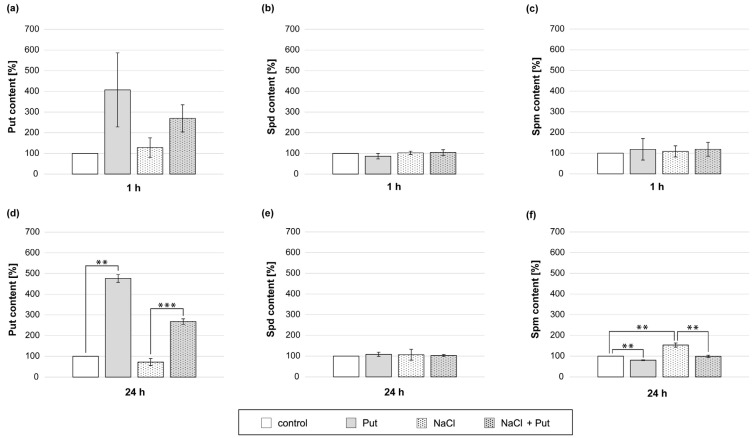
Endogenous PA levels in roots of cucumber seedlings treated with 1 mM Put and/or 120 mM NaCl for (**a**–**c**) 1 h and (**d**–**f**) 24 h. The average value of control plants, based on the results of individual experiments, was 42.24 ± 16.04 µg Put × gFW^−1^, 79.56 ± 9.59 µg Spd × gFW^−1^ and 6.03 ± 1.68 µg Spm × gFW^−1^ for 1 h (**a**–**c**) and 24.23 ± 4.25 µg Put × gFW^−1^, 59.12 ± 10.31 µg Spd × gFW^−1^, and 3.81 ± 0.27 µg Spm × gFW^−1^ for 24 h (**d**–**f**). Significance was evaluated at: *p* < 0.05 (**); *p* < 0.001 (***).

To investigate the connection between exogenous Put application and PA metabolism in non-stressed and salt-stressed cucumber roots, the endogenous PA content was measured. The content of endogenous PAs in roots of cucumbers treated with Put and/or NaCl for 1 h was not significantly changed (Figure 8a–c). However, the modulation in PA content was noticeable during longer, 24 h treatments (Figure 8d–f). In response to the Put application for 24 h, endogenous Put content in roots increased by about 3-fold compared to control plants (Figure 8d). In roots treated with NaCl, the level of endogenous Put in roots was reduced by about 30% compared with non-stressed control (Figure 8d). Treatment of plants with Put, together with salt, caused an increase in endogenous Put content in roots approximately 4-fold compared with the NaCl treatment (Figure 8d). In plants treated with Put, endogenous Spm content was reduced by about 20% compared to control (Figure 8f). On the other hand, the treatment of seedlings with NaCl increased endogenous Spm content in roots by about 50% compared to non-stressed plants. However, 24 h incubation of plants in the presence of Put and NaCl significantly lowered the endogenous content of Spm in the roots in relation to plants treated with salt only (Figure 8f).

## 4. Discussion

Nitric oxide is an important signaling molecule participating in plant response to salt stress. It can reduce the adverse effects of salinity, e.g., by improving plant water management and ion homeostasis, induction of osmolytes and osmoprotectant accumulation, and reducing ROS accumulation [9,69]. 

Over the years, the number of reports indicating the participation of PAs in NO production has increased [15,44]. In the present study, it was demonstrated that, among the three PAs tested, only exogenous Put significantly stimulated NO generation in cucumber roots (Figure 1 and Figure S1). The observed changes may be related to plant species and possibly PA absorption. As small, aliphatic polycations, PAs easily bind to the components of cell walls. Put exhibits less positive charges; therefore, it is much easier than it is for Spd or Spm to move into cells [15,70]. Furthermore, some research has supported a thesis that PAs can regulate NO production in plants subjected to abiotic stresses. In tomato roots subjected to 75 mM NaHCO_3_ for 5, 10, and 15 h, the NO production increased, and the application of PAs elevated NO biosynthesis under Na^+^ toxicity and oxidative stress induced by NaHCO_3_ [71]. Similarly, in tomato leaves exposed to cold stress, Spd and Spm treatment significantly stimulated NO production [35]. Based on these results, it was suggested that exogenous PAs, through the modulation of NO levels in plant organs, may induce plant tolerance to stresses [35,36,45]. 

In our previous studies, salt stress-induced NO production was demonstrated [29,53]. Therefore, in this study, the impact of Put on NO level in cucumber seedling roots subjected to 120 mM NaCl was investigated. According to the previous suggestions of Mur et al. [58], two independent and commonly used methods of NO detection were performed—the procedure measuring NO-related DAF-2 DA green fluorescence and the colorimetric method with the Griess reagent [59]. Salt stress caused by 120 mM NaCl stimulated NO production in the roots of cucumber seedlings (Figure 1). However, exogenous Put present in the environment along with salt stress regulated salt-induced NO generation in a time-dependent manner. During short-term (1 h) salt stress, exogenous Put reduced NO production. On the other hand, in the longer (24 h) salt stress treatment, Put treatment led to a further increase in NO biosynthesis in cucumber roots (Figure 1). The dual regulatory role of exogenous Put in NO production may be related to the mechanism of plant response to salt stress. It is possible that during the short-term salt stress, exogenous Put may participate in NO homeostasis maintenance by reducing the level of this molecule. In contrast, during a longer period of salt stress, exogenous Put may boost NO biosynthesis to induce plant responses to stress. A slight but positive impact of exogenous Put on plant growth parameters and RWC (Figure S3) may support the role of this PA in cucumber response to salt stress.

In plants, Put production can occur by two pathways, including the ADC enzyme and ODC enzyme [72]. The application of ADC and ODC inhibitors counteracted the increase in NO levels in the roots of cucumbers exposed to salt stress (Figure 2). It indicated that endogenous Put, produced with the participation of ODC and ADC enzymes, can take part in enhanced NO production in cucumber roots during salinity.

Polyamines may modify two key pathways of NO biosynthesis: the reductive pathway with the participation of NR and the oxidative pathway with NOS-like activity [35,36]. The application of sodium tungstate, a commonly used NR inhibitor, significantly prevented NO production in the roots of cucumber seedlings treated with salt, indicating the involvement of NR and/or other molybdoenzymes in this process (Figure 3). Short-term (1 h) salinity enhanced total NR activity, pointing to NR-dependent NO production in examined conditions (Figure 4a). On the other hand, a longer duration of salt stress (24 h) resulted in a strong decrease in total NR activity (Figure 4d), which suggests that other molybdoenzymes, such as XOR, AO, or ARC, may also participate in higher NO production in plants under salt stress. Further results showed that exogenous Put can modulate NR-dependent NO production during salt stress. Exogenous Put decreased NO biosynthesis in cucumbers exposed to short-term (1 h) salt stress (Figure 1), which corresponded to a reduced total NR activity in these conditions (Figure 4a). On the other hand, during longer (24 h) salt treatment, exogenous Put not only induced NO level in roots but also diminished the inhibitory effect of salinity on the total NR activity. Similar to this, the application of other PAs (Spd and Spm) during chilling stress has further elevated NO production and NR activity in tomato leaves [35]. The positive influence of exogenous PAs on total NR activity was also demonstrated in tomato leaves exposed to heat stress, rice seedlings subjected to NaF, and creeping bentgrass leaves under drought conditions [73,74,75]. The regulatory role of Put in relation to total NR activity may have a dual significance. Firstly, PAs can control NR activity and, consequently, NO levels under stress conditions [35,36]. Therefore, it is possible that exogenous Put, via modulation of NR-dependent NO production, may play a role in the maintenance of NO homeostasis during short-term salt stress and may participate in triggering NO-related signaling pathways that induce plant defense mechanisms during longer salinity. Secondly, NR is an essential enzyme involved in nitrogen metabolism. Therefore, the modulation of NR activity by PAs during salinity can improve the assimilation of this macroelement and may be another important mechanism in PA-induced plant stress tolerance [37,75].

The NR activity is regulated at the post-translational level through phosphorylation, SUMOilation, S-nitrosylation, and nitration [60,76,77,78]. Among these, reversible NR phosphorylation is the best understood [60,79]. Changes in the phosphorylation/dephosphorylation level of NR are noticeable during the analysis of actual NR activity and NR activation state. Actual and total nitrate reductase activities allow us to determine the NR activation state, which shows the level of unphosphorylated NR in relation to the whole enzyme pool (actual/total ratio) (Figure S1c) [21]. In the roots of cucumbers subjected to salt stress for 1 h, the higher actual NR activity was accompanied by an enhanced NR activation state (Figure 4b,c), which can be interpreted as an increase in the dpNR form in the enzyme pool. In contrast, exposure of cucumber seedlings to salt stress for 24 h resulted in a reduction of the actual NR activity in roots and decreased NR activation state (Figure 4e,f), indicating a decrease in the dpNR form in the total enzyme pool. These changes illustrate the time-dependent regulation of NR activity by reversible phosphorylation/dephosphorylation under salt stress conditions. In a previous study, Reda et al. [29] confirmed that NR dephosphorylation occurs in cucumber roots in response to long-term 50 mM NaCl treatment. Furthermore, it was assumed that total NR activity parameters, such as actual NR activity and NR activation state, could also be important in the identification of NR-dependent NO production in plants [29]. During 1 h salt stress treatments, the activation state of the NR was stimulated, which means that the amount of the active dpNR form that can produce NO increased. However, during plant exposure to salt stress for 24 h, the activation state of NR was inhibited, indicating that the active dpNR form decreased, which could reduce NO biosynthesis. It was shown that exogenous Put reduced the actual NR activity and NR activation state in the roots of cucumbers exposed to short-term salinity (Figure 4b,c) and enhanced these parameters in roots during longer salinity (Figure 4e,f). These Put-induced post-translational modifications of NR activity by reducing (1 h of stress) or increasing (24 h of stress) the active dpNR form may be responsible for the changes in NR-dependent NO production observed in the presence of exogenous Put during salt stress. 

Other enzymes that are involved in NO production in plant roots are NiNOR and PM-NR [18,29,30]. It was shown that PM-NR may participate in NO production in plant roots exposed to mild (50 mM NaCl) salt stress. However, in the present work, salt stress caused by 120 mM NaCl gradually reduced PM-NR activity in cucumber roots (Figure 5). A decreased PM-NR activity may lead to lower apoplastic NO_2_^−^ levels in plant roots, which may inhibit NO production through NiNOR [29]. There may be two reasons for the negative regulation of PM-NR activity during salt stress. First, one of the effects of salinity is the induction of oxidative and nitrosative stress [36]. Consequently, the protein–lipid interactions are disturbed at the membrane level, which has an adverse effect on the plasma membrane integrity and activity of PM-bound enzymes [80]. Also, the enzymatic activity of PM-NR is strongly associated with the presence of the substrate, NO_3_^−^ ions, and that level is reduced during salt stress [29,30]. However, exogenous Put diminished the inhibitory effect of salinity on PM-NR activity during longer (24 h) salt stress, indicating a protective effect of Put on the PM-bound NO production system. The effect of Put on PM-NR activity was different during non-stress and stress conditions. Recent studies showed that PAs conjugated to plasma membranes can increase plant tolerance to abiotic stress by stabilizing the membrane structure [81]. Exogenous PAs may also positively affect the level of NO_3_^−^ ions in plant organs during abiotic stresses [37]. Accordingly, the influence of exogenous Put on PM-NR activity may be associated with the protective function of PAs on biological membranes and/or the restoration of ion homeostasis under stress conditions.

Modifications of total NR activity, and possibly PM-NR activity, can reflect changes in the expression of genes encoding this enzyme. This relationship has been shown in *Arabidopsis* roots, where a positive correlation between total NR activity and *NIA2* expression during salt stress was noted [82]. Previous studies have demonstrated that NR activity is precisely regulated at the genetic level through modifications of *CsNR* genes in response to different durations and intensities of salt stress [29,52]. Our analysis revealed a significant down-regulation of *CsNR1* expression and up-regulation of *CsNR3* expression in cucumber roots in response to a 1 h salt treatment (Figure 6a,c). On the other hand, longer (24 h) exposure of plants to salt stress caused a down-regulation of *CsNR1–3* expression (Figure 6d–f). Therefore, we suggest, with great caution, that the CsNR3 isoform may play an essential role in NR-mediated NO production at the early stages of moderate salt stress. On the other hand, lower expression of *CsNR1–3* could be responsible for strong inhibition of total NR activity and possibly PM-NR activity in cucumber roots exposed to 24 h salinity (Figure 4d and Figure 5b). On the other hand, some previous studies presented the positive impact of exogenous PAs on NR genes in plants subjected to abiotic stresses [73,74]. Results in this work demonstrated that under non-stress conditions, exogenous Put added to the environment for 1 h modulated *CsNR1* and *CsNR3* expression in cucumber roots similarly to salt (Figure 6a,c). However, under salinity, the impact of exogenous Put on *CsNR1–3* expression in cucumber roots changed significantly. Exogenous PA during 1 h salt stress upregulated the expression of *CsNR1* and *CsNR2* and down-regulated the expression of *CsNR3*, which was accompanied by reduced total NR activity (Figure 4a). It indicates that, in addition to the post-transcriptional level, exogenous Put may also affect the activity of NR and further NR-dependent NO biosynthesis at the genetic level by regulating the expression of genes encoding the enzyme. Furthermore, it should be noticed in this case that an increase in *CsNR1* and *CsNR2* gene expression does not always correlate with protein activity. Changes in gene expression do not always directly correspond to protein levels. Cellular protein abundance is the result of a dynamic balance between various processes, from the transcription, processing, and degradation of mRNAs to translation, localization, modification, and finally, the programmed destruction of the proteins themselves [83,84].

Unlike NR-dependent reductive NO biosynthesis, NOS-like-dependent NO production has been described during plant response to salt stress, heavy metal stress, and drought [45,85]. The participation of NOS-like activity in the NO generation in cucumber roots during salt stress was confirmed with L-NAME, the potent inhibitor of animal NOS, which effectively counteracted the increase in NO level in the roots of salt-stressed plants (Figure 3). However, the NOS-like activity was just slightly modulated in the roots of cucumber seedlings subjected to salt stress for 1 h (Figure 7a). Exogenous PAs may affect NO production through NOS-like activity. The regulatory role of exogenous Spd and Spm was shown during NOS-like dependent NO production in plants exposed to cold stress [35]. Exogenous Put induced NO production via participation of NOS-like activity in *Anthurium andraeanum* during chilling stress [86]. Nevertheless, the results in the present work do not confirm what is mentioned above. 

Under salt stress conditions, the endogenous PA content changes in plant organs, which is often linked with plant tolerance to stress [36,75]. Our analysis showed that the PA pool in cucumber roots was modulated in response to 24 h NaCl treatment, indicating that PA metabolism is regulated by salt stress. During 24 h salt treatment, cucumber roots accumulated a lower content of endogenous Put and a higher content of exogenous Spm (Figure 8d,f), which might be attributed to the metabolic conversion of Put to Spm and/or PA translocation from the roots to shoots [36]. On the contrary, exogenous Put treatment had the opposite effect on these two endogenous PA contents in cucumber roots exposed to salt stress for 24 h (Figure 8d,f). Endogenous PAs can participate in the post-translational regulation of NR activity. In the presence of Mg^2+^, Spd, or Spm, pNR interacts with 14-3-3 protein, resulting in the inhibition of NR activity [21,87]. Obtained data suggest that during longer salt stress (24 h), the actual NR activity and NR activation state may be reduced due to endogenous Spm accumulation accompanied by Put content reduction. However, the application of exogenous Put resulted in higher endogenous Put content and reduced endogenous Spm content. It may provoke unfavorable conditions to the interaction of pNR and 14-3-3 protein and may restore NR activity, leading to boosted NO production in plants subjected to longer salt stress.

**Figure 9:**
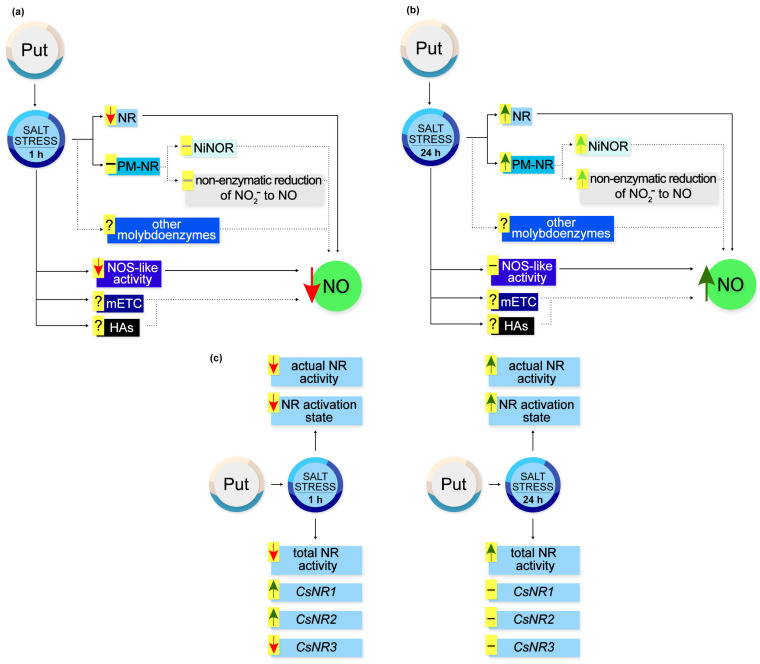
Effect of exogenous Put on NO production and NR activity in roots of cucumber seedlings exposed to salt stress. (**a**) During short-term (1 h) salt stress exogenous Put diminished NR-dependent NO production in cucumber roots. At the same time, exogenous Put reduced NO generation through NOS-like activity. However, exogenous Put did not modulate the activity of PM-NR, which may not modify NO biosynthesis via the NiNOR enzyme and the non-enzymatic pathway in the apoplast of cucumber roots. It should be noted that the observed changes in the NO level might result from the possible influence of exogenous Put on other pathways of NO production in cucumber roots during salt stress. (**b**) Under longer (24 h) periods of salt stress, exogenous Put triggered the stimulatory effect of NaCl on NO production in cucumber roots. Exogenous Put enhanced the total NR activity and PM-NR activity. The stimulation of PM-NR activity may increase NO generation in the apoplast of plant roots. Exogenous Put might affect other possible pathways of NO biosynthesis during longer salt stress. (**c**) Time-dependent regulation of NR activity at the genetic and post-translational level by exogenous Put in cucumber roots subjected to salt stress. Markings: **↑**, stimulation; **↑**, possible stimulation; **↓**, inhibition; **−**, no changes; −, possible no changes. HA, hydroxylamine; mETC, mitochondrial electron transport chain; NiNOR, plasma membrane-bound nitrite-NO reductase; NOS-like activity, nitric oxide synthase-like activity; NR, nitrate reductase; PM-NR, plasma-membrane nitrate reductase; Put, putrescine.

Based on the obtained results, a schematic model illustrating the action of exogenous Put during NO production and regulation of NR activity in cucumber roots in response to salt stress is illustrated in Figure 9.

## 5. Conclusions

Exogenous Put can play a regulatory role in controlling NO production in cucumber roots during the plant response to salt stress. During salinity, this PA may maintain NO homeostasis by managing the activity of selected NO biosynthesis pathways, especially NR activity. This control undergoes at the genetic and post-translational level in a time-dependent manner. Under short-term (1 h) salt stress, exogenous Put decreased NO level, influencing the two main NO production pathways, NR and NOS-like activity. It diminished NR-dependent NO production by reducing both the total NR activity and the level of the active dpNR form of the enzyme. Exogenous Put also downregulated *CsNR3* expression, which might be related to NO biosynthesis. In addition to NR, exogenous Put also reduced NOS-like activity in cucumber roots. During longer (24 h) salt exposure, exogenous Put may maintain a NO-dependent plant response to salinity. It boosted NO production by restoring NR and PM-NR activity. First, this PA alleviated the adverse effects of stress on total NR activity and increased the level of the dpNR form of the enzyme. Second, exogenous Put reduced the inhibitory effect of salinity on PM-NR activity, probably by increasing NO biosynthesis via NiNOR. The role of endogenous Put in salt-induced NO production was confirmed using the ADC and ODC inhibitors. 

## Data Availability

The data presented are available in this manuscript and Supplementary Materials.

## Supplementary Materials

The following supporting information can be downloaded at: https://www.mdpi.com/article/10.3390/metabo13091030/s1, Figure S1: Graphical illustration of total and actual NR activities; Figure S2: NO production in roots of cucumber treated with 1 mM Spd and 1 mM Spm for 1 h and 24 h; Figure S3: Growth parameters and relative water content (RWC) in cucumber seedlings treated with 1 mM Put and/or 120 mM NaCl for 1 h and 24 h; Table S1: Activities of ADH and PEPC in the plasma membrane fraction. Ref. [88] is cited in Supplementary Materials.
