# Supplementary material for: Exogenous Putrescine Modulates Nitrate Reductase-Dependent NO Production in Cucumber Seedlings Subjected to Salt Stress
Source: Metabolites. 2023 Sep 21;13(9):1030. doi: 10.3390/metabo13091030 (PMC10535175; doi:10.3390/metabo13091030)
Supplement: Supplementary file 1 [file metabolites-13-01030-s001.zip › metabolites-2607549-supplementary.pdf]

Supplementary Materials

Exogenous putrescine modulates nitrate reductase-dependent NO production in cucumber seedlings subjected to salt stress

Natalia Napieraj, Małgorzata Janicka, Beata Augustyniak and Małgorzata Reda

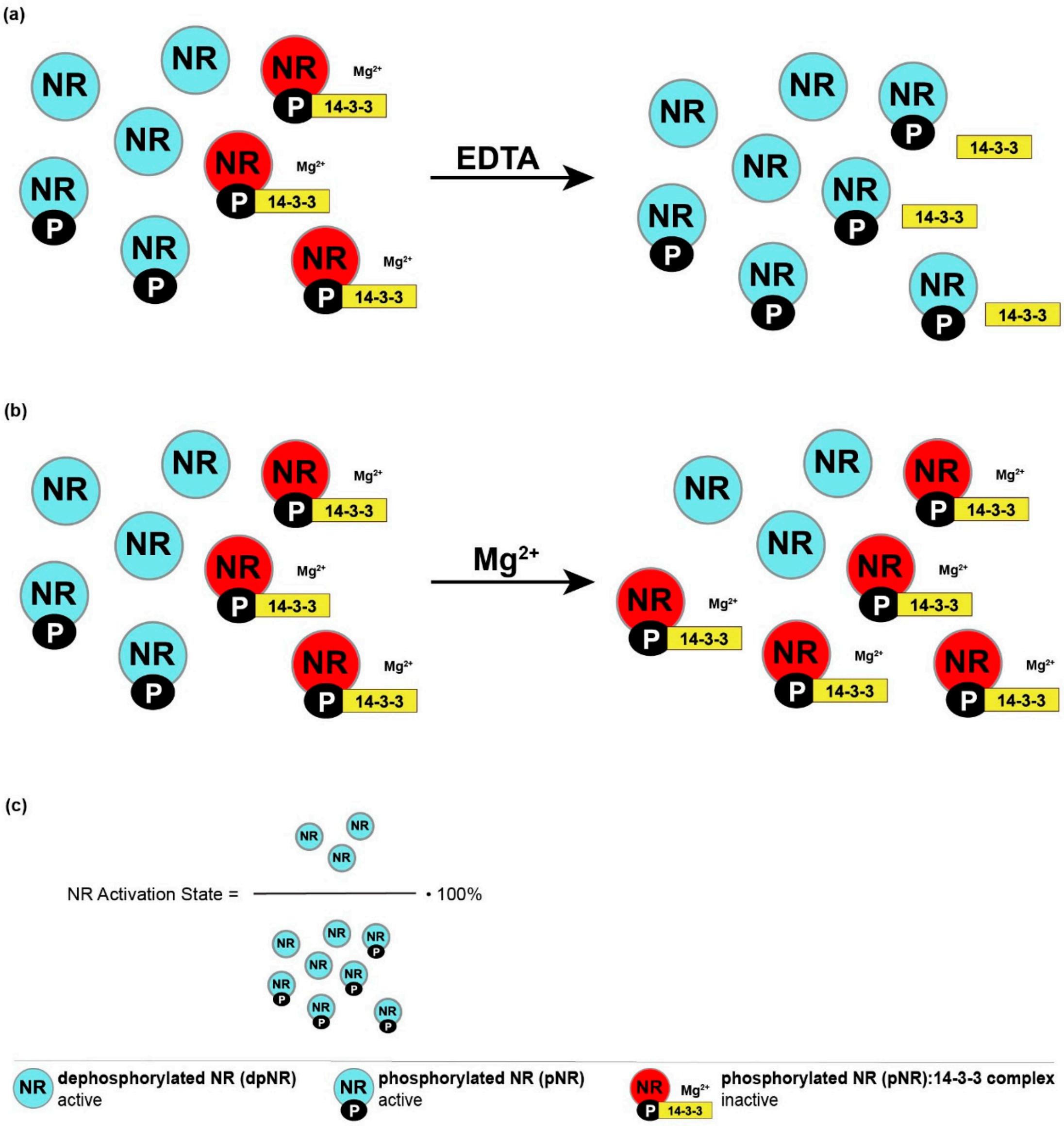

Figure S1: Graphical illustration of total and actual NR activities. NR occurs in three states. Free, unphosphorylated NR is responsible for NO_3^- reduction. When NR occurs in phosphorylated form (pNR), the enzyme is active until the 14-3-3 protein recognizes and binds to pNR in the presence of divalent cations (e.g. Mg^{2+}) or PAs. Therefore, the third form of NR is the inactive pNR:14-3-3 complex[Chamizo-Ampudia et al. 2017]. (a) To determine the total NR activity, EDTA (Mg^{2+} chelator)

was added into the reaction mixture. Under these conditions, inactive pNR:14-3-3 complexes were not present, and the activity of both the unphosphorylated and phosphorylated forms of NR was detected. (b) NR activity was measured in the presence of Mg2+ ($MgCl_2$) in the reaction mixture. Under these conditions, the phosphorylated enzyme pool (pNR) interacts with the 14-3-3 protein and forms inactive pNR:14-3-3 complexes. Hence, actual NR activity describes only the activity of the unphosphorylated form of NR. Actual and total nitrate reductase activities allow to estimate the NR activation state, which shows the level of unphosphorylated NR in relation to the whole enzyme pool (actual/total ratio) expressed as percentage.

References:

Chamizo-Ampudia A, Sanz-Luque E, Llamas A, Galvan A, Fernandez E, 2017, Nitrate reductase regulates plant nitric oxide homeostasis. Trends in Plant Science 22: 163-174

Table S1: Activities of ADH and PEPC in the plasma membrane fraction.

**Enzyme**	**Activity in the cytosol fraction** [μmol NADH × min^{-1} × cm^{-3}]	**Activity in the plasma membrane fraction** [μmol NADH × min^{-1} × cm^{-3}]
ADH	0,0291 ± 0,0057	0,0035 ± 0,0023**
PEPC	0,0600 ± 0,0044	0,0007 ± 0,0007***

Significance was evaluated at: p<0,1 (*); p<0,05 (**); p<0,001 (***).

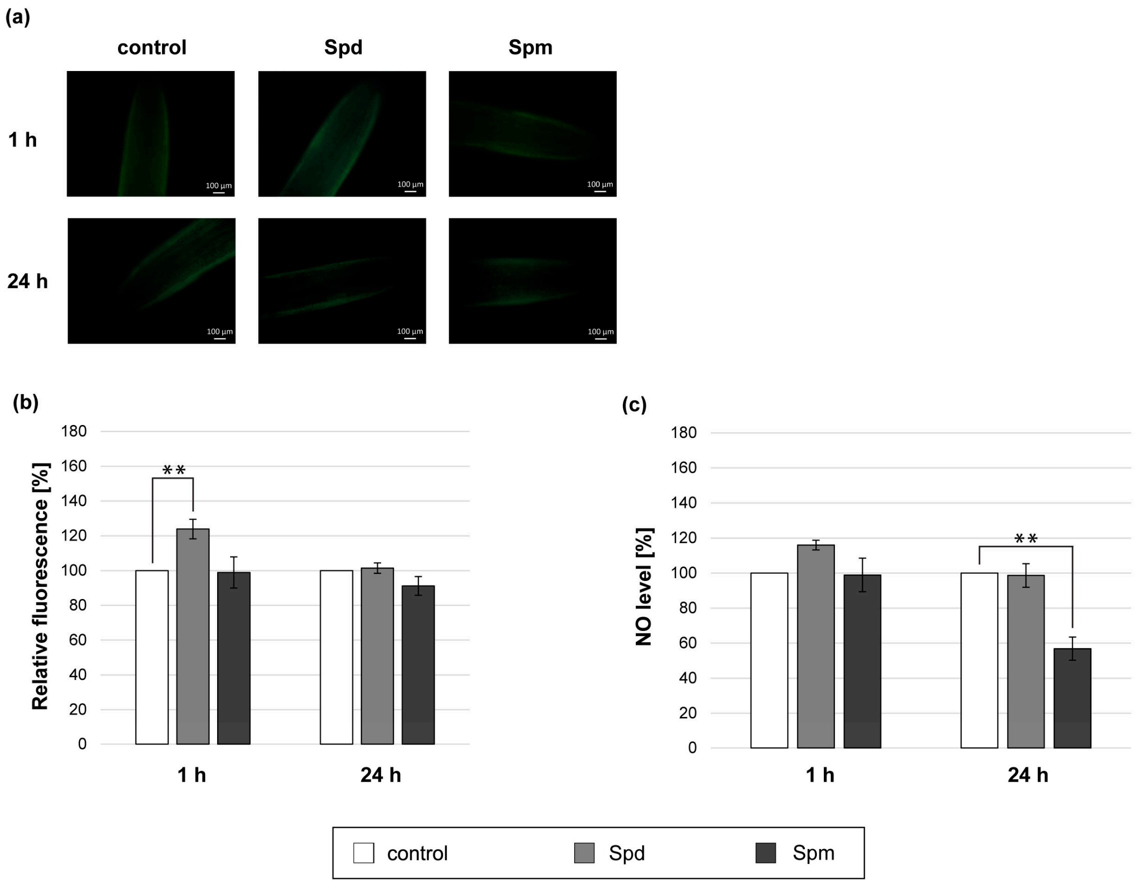

Figure S2: NO production in roots of cucumber treated with 1 mM Spd and 1 mM Spm for 1 h and 24 h. (**a**) Bio-imaging of NO generation in root apical segments of cucumbers using a fluorescent method with DAF-2DA dye. Bar, 100 µm. (**b**) Relative efficiency of Spd and Spm for NO release from root apical segments of cucumber seedlings. (**c**) NO level in cucumber roots measured colorimetrically using the Griess reagent. The average value of untreated (~~Spd, Spm~~control) plants was 9.69 ± 1.76 nmol NO_2^- × gFW^{-1}. Results are means ± SD from at least three independent experiments with each experiment performed in three replicates. Significance was evaluated at: p<0,05 (**).

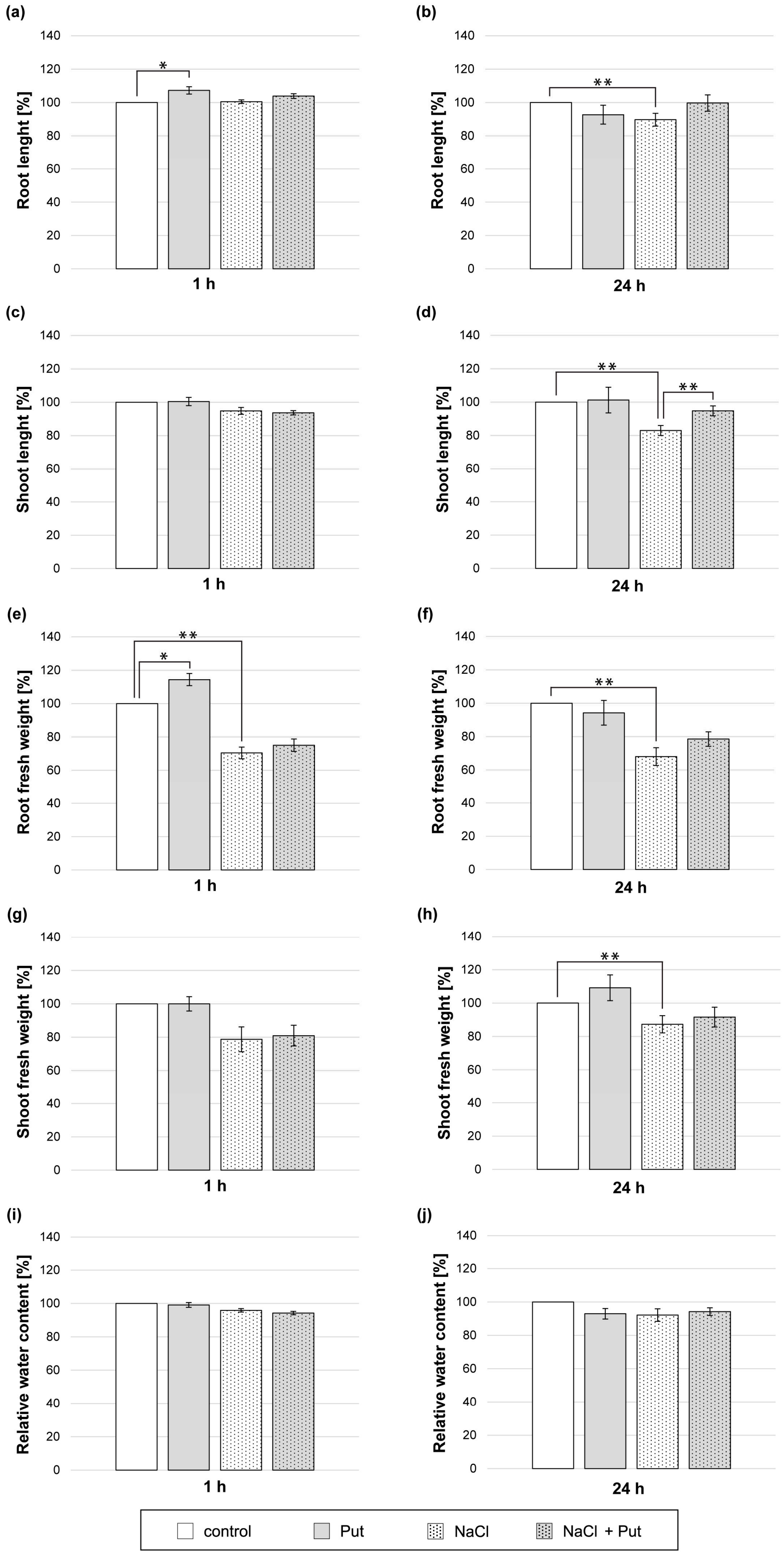

(a)
Root lenght [%]
1 h
(b)
Root lenght [%]
24 h
(c)
Shoot lenght [%]
1 h
(d)
Shoot lenght [%]
24 h
(e)
Root fresh weight [%]
1 h
(f)
Root fresh weight [%]
24 h
(g)
Shoot fresh weight [%]
1 h
(h)
Shoot fresh weight [%]
24 h
(i)
Relative water content [%]
1 h
(j)
Relative water content [%]
24 h
control
Put
NaCl
NaCl + Put

Figure S3. Growth parameters and relative water content (RWC) in cucumber seedlings treated with 1 mM Put and/or 120 mM NaCl for 1 h and 24 h. The average value of untreated (control) plants was 3.32 ± 0.15 cm × plant $^{-1}$ for shoot length, 11.26 ± 0.21 cm × plant $^{-1}$ for root length, 0.089 ± 0.004 g × plant $^{-1}$ for fresh shoot weight, 0.122 ± 0.006 g × plant $^{-1}$ for fresh root weight and 0.8649 ± 0,0127 for RWC. Data are means ± SD from at least three independent experiments with each experiment performed in eight replicates. Significance was evaluated at: $p<0,1$ (*); $p<0,05$ (**).
